# Expanded phenotype in a patient with spastic paraplegia 7

**DOI:** 10.1002/ccr3.1109

**Published:** 2017-08-24

**Authors:** Jennifer Gass, Patrick R. Blackburn, Jessica Jackson, Sarah Macklin, Jay van Gerpen, Paldeep S. Atwal

**Affiliations:** ^1^ Center for Individualized Medicine Mayo Clinic 4500 San Pablo Road South Jacksonville Florida 32224 USA; ^2^ Department of Clinical Genomics Mayo Clinic 4500 San Pablo Road South Jacksonville Florida 32224 USA; ^3^ Department of Neurology Mayo Clinic 4500 San Pablo Road South Jacksonville Florida 32224 USA

**Keywords:** Ataxia, palatal tremor, paraplegin, spastic paraplegia 7, *SPG7*

## Abstract

Hereditary spastic paraplegia is a group of clinically and genetically heterogeneous neurodegenerative disorders, often characterized by weakness and spasticity in the lower limbs. In our study, we describe a spastic paraplegia type 7 patient with an expanded phenotype who was diagnosed after the discovery of pathogenic variants in *SPG7*.

## Introduction

Hereditary spastic paraplegia (HSP) is a group of neurodegenerative disorders characterized by progressive bilateral lower limb weakness and spasticity. In addition, HSP patients may also develop hyperreflexia in the arms, dysphagia, ataxia, nystagmus and strabismus, loss of hearing, motor and sensory neuropathy, and amyotrophy 1. According to OMIM, over 80 different genetic loci have been identified to cause HSP through autosomal dominant, autosomal recessive, or X‐linked modes of inheritance (https://omim.org/) 2, 3. Proteins encoded by these genes have a wide range of functions, including myelin formation, axonal transport, endoplasmic reticulum morphology, and mitochondrial function 2. The most common pathological feature of HSP is selective degeneration of the corticospinal tract and fasciculus gracilis, suggesting that these proteins are somehow involved in the survival of specific neurons 4. Postmortem studies have also concluded that in some cases, degeneration may extend into the midbrain to include the pons, medulla, cerebral peduncles, or beyond 2.

Spastic paraplegia type 7 (SPG7, MIM: 607259) is an autosomal recessive HSP caused by various pathogenic variants (i.e., missense, nonsense, splice site, frameshift, and deletion or duplication) in the spastic paraplegia 7 gene (*SPG7*, MIM: 602783) 1, 5, 6. *SPG7* encodes for paraplegin, a nuclear‐encoded mitochondrial ATPase 7. Studies in knockout (*Spg7* ^*−/−*^) mice revealed that loss of SPG7 triggers ATPase deficiency and mitochondrial dysfunction 8. Upon further examination of these mice, it was clear that the mitochondrial dysfunction was a result of paraplegin loss, leading to accumulation of abnormal proteins and impairment of anterograde and retrograde transport which caused axonal degeneration. Furthermore, in patient muscle biopsies, similar mitochondrial impairment was observed 9. Additional studies also report how paraplegin functions to degrade misfolded proteins and regulate ribosome assembly 10, 11.

Clinically, patients with the SPG7 subtype may present with a pure form of HSP and display the aforementioned symptoms 12. On the other hand, complex forms also occur and include additional symptoms such as cortical and cerebellar atrophy, optic neuropathy, and peripheral neuropathy. In our case study, we describe a patient with a compound heterozygous *SPG7* variant and previously unreported symptoms, involving palatal tremor and macrocephaly.

## Case History

The patient, a 46‐year‐old man, initially presented at our clinic with progressive difficulties with gait and balance. Onset of these symptoms first appeared 5 years prior and evolved to include changes in speech, frequent choking while eating and increased occurrence of falls. The patient did not report similar findings in additional family members and remarked that as a child he suffered from speech delay but took normal classes during high school. Recently, his wife explained that she noticed a gradual decrease in cognition, revealing that his full‐scale IQ has dropped from 115 to 85 over the last 20 years. Due to his cognitive decline, he was unable to keep employment at his job of 20 years.

### Neurological investigations

A comprehensive neurological examination revealed several salient findings, including mild primarily ataxic dysarthria, palatal tremor, and ataxic saccades. In a visual fixation test, microsquare wave jerks were also evident. Tandem‐walking was impaired. The patient's casual gait revealed an erect posture with a normal arm swing; however, his base was wide and bilateral circumduction was present. Postural reflexes were impaired on pull testing, and Romberg's sign was absent. While standing, there were no subjective or palpable lower extremity movements. Appendicular ataxia was present, including dysdiadochokinesia and moderate dyssynergia on heel‐skin testing. No limb weakness was observed, although the patient had pathologically brisk myotatic stretch reflexes at the knees. His head circumference measured above the 97th percentile at 63.5 cm diagnostic of macrocephaly, and a recent brain MRI revealed only mild cerebellar atrophy. Specifically, the white matter was normal.

## Genetic Testing

Prior to visiting our clinic, the patient was tested for disease‐causing genetic variants using a comprehensive ataxia panel test from Athena diagnostics. Sequencing results were negative for pathogenic disease‐causing variants in all gene screened in this panel (i.e., ADCK3, AFG3L2, ANO10, APTX, ATM, ATN1, ATXN1, ATXN10, ATXN2, ATXN3, ATXN7, ATXN8OS, CACNA1A, CACNB4, EEF2, FGF14, FLVCR1, FXN, GRM1, ITPR1, KCNA1, KCNC3, KCND3, MRE11A, MTPAP, PDYN, POLG, PPP2R2B, PRKCG, SACS, SETX, SIL1, SLC1A3, SPTBN2, SYNE1, SYT14, TBP, TDP1, TGM6, TTBK2, TTPA, VAMP1). To further examine this patient for any underlying genetic disorders, we submitted samples from the patient, his mother, and his father for whole exome sequencing at GeneDx. Using genomic DNA, exonic regions and flanking splice junctions were sequenced and aligned to reference sequences on the human genome using standard methods. Results revealed compound heterozygous variants in *SPG7* involving a p.Ala510Val (c.1529C>T) pathogenic variant and a p.Arg485_Glu487del (c.1454_1462del) variant. The c.1529C>T variant inherited from his mother is a common pathogenic variant identified in patients with autosomal recessive SPG7. Our patient also inherited a different *SPG7* variant, c.1454_1462del, from his father. The presence of these bi‐allelic pathogenic variants is consistent with a diagnosis of SPG7, a rare neurodegenerative disorder depicted by his presenting symptoms.

## Discussion

Spastic paraplegia type 7 onset typically occurs in adulthood, but certain cases have been reported as early as 11‐year old and late as 72‐year old 13. The majority of SPG7 affected individuals have proximal or widespread weakness in their legs in addition to various other symptoms (e.g., ataxia, intellectual disability, optic nerve atrophy, and cervical dystonia) 2. Furthermore, when comparing previous studies and our own case, there is no correlation between a specific genotype and a patient's phenotype. The variants our patient acquired have previously been described as causing both pure and complex HSP 3. Moreover, we are the first to report macrocephaly and palatal tremor in a patient with SPG7, which represents an expansion of the clinical phenotype for this disorder. Due to his presentation, Alexander's disease was a strong consideration: However, the patient was not found to have a pathogenic variant in glial fibrillary acidic protein (*GFAP*) 14, 15. Interestingly, ataxias and spastic paraplegias are part of a disease continuum and often have overlapping phenotypes and genetic causes. In fact, pathogenic variants in *SPG7*,* SYNE1,* and *PNPLA6* are known to cause both inherited ataxias and HSP 16. Panel testing for hereditary ataxias was uninformative in our case, making whole exome sequencing essential for the correct diagnosis. Knowing this, future ataxia or HSP panels should be designed to include genes that are involved in both diseases.

At this time, there is no treatment for SPG7. Our patient is currently being treated for sleep apnea with Continuous Positive Airway Pressure therapy and will be monitored for any remarkable changes due to his SPG7. In conclusion, our case report emphasizes the importance of genetic testing when diagnosing rare neurodegenerative disorders and expands the phenotypic spectrum of SPG7. Along with Alexander's disease, SPG7 should be considered in the setting of PAPT.

## Ethics Approval and Consent

Diagnosis, treatment, and counseling were performed following the principles of medical ethics. The authors have obtained proper consent to publish data collected from this patient. All forms have been properly signed and are available upon request.

## Conflict of Interests

There are no conflict of interests to report. All authors have approved the content of the manuscript.

## Authorship

JG: organized and wrote the first draft. PB: involved review and critique. JJ: performed genetic counselor, review, and critique. SM: performed genetic counselor, review, and critique. J van Gerpen: performed neurologist, project execution, review, and critique. PSA: performed medical geneticist, project execution, design, review, and critique.
